# Prevalence of lower urinary tract symptoms in nurses and civil servants working at a hospital: a cross-sectional study

**DOI:** 10.4314/ahs.v21i1.29

**Published:** 2021-03

**Authors:** Gulsah Kok, Semra Kocaoz, Gulten Guvenc, Aygul Akyuz

**Affiliations:** 1 University of Health Sciences Turkey, Gulhane Faculty of Nursing, Obstetrics and Gynecology Nursing Department, Etlik/Ankara, Turkey; 2 Nigde Omer Halisdemir University, Nigde Zubeyde Hanim School of Health, Department of Obstetrics and Gynecology Nursing, Derbent Campus, 51200, Nigde/ Turkey; 3 Demiroglu Bilim University, Florence Nightingale Hospital School of Nursing, Istanbul /Turkey

**Keywords:** Lower urinary tract symptoms, prevalence, nurses, civil servants

## Abstract

**Background:**

Lower urinary tract symptoms (LUTS) are common in women and can interrupt daily living activities of the individuals. The study aimed at determining the LUTS prevalence and the influencing factors in nurses and civil servants working at a hospital.

**Methods:**

This cross-sectional and descriptive study was conducted with 158 female nurses and 105 female civil servants. The data were obtained with a data collection form and the Bristol Female Lower Urinary Tract Symptoms-Short Form.

**Results:**

This paper exposes that the prevalence of at least one LUTS was 94.2% in nurses and 97.1% in civil servants. The most common LUTS symptoms of nurses and civil servants were urgency (60.1% nurses, 81.9% civil servants) and urge incontinence (59.5% nurses, 81.9% civil servants). Nurses (60.8%) expressed significantly higher rates of having inadequate time going to the toilet due to their work conditions compared to the civil servants (41.9%) (p<0.05). BFLUTS-SF scores in terms of age, BMI, parity, having cesarean and vaginal delivery and urinary incontinence in their previous pregnancies were compared between two groups, statistically significant differences were found (p<0.05).

**Conclusion:**

Workplace conditions of the health workers should be reorganizing to have healthy urinary habits for preventing them from the development of LUTS.

## Introduction

Lower urinary tract symptoms (LUTS) are perceived as “a disturbance or change in the present situation” by the individuals, their caregivers or spouses. LUTS are classified under three main headers as storage, voiding and post micturition symptoms.^1^ LUTS are common in females.^2^,^3^ The prevalence of LUTS has been reported to vary between 27.78% and 94.3% in previous studies.^4^,^5^,^6^ Many risk factors are thought to play a role in the development of LUTS that is seen at different prevalence rates worldwide.^7^,^8^,^9^ Advanced age^7^, increased autonomous nervous system activity, detrusor sensitivity, endothelial dysfunction, chronic inflammation, and oxidative damage are also reported to play a role in LUTS pathogenesis in females.^8^ Chronic diseases and such as diabetes, hypertension, and asthma also influence LUTS development in females.^9^

LUTS affect the quality of life of women negatively.^2^,^3^,^10^ LUTS can cause emotional stress, decrease the quality of life, interrupt daily living activities in females^10^ and also decrease the work quality of the individuals.^11^ Various workplace environment factors such as inappropriate physical conditions of the toilet at work, not finding enough time to urinate at workplace, the possibility of dangerous work and accidents, time pressure, working in an inappropriate position for long periods, and carrying heavy loads were seen to influence the development of UI (urinary incontinence) and LUTS.^12^,^13^ Nurses also work under many risky conditions that cause the development of LUTS as mentioned in the literature.^14^ Various studies have emphasized the effect of work conditions on the increased risk of LUTS development.^13^,^14^

This study was aimed to identify LUTS prevalence and the influencing factors in female nurses and civil servants working at a hospital. It was also tried to determine whether the factors influencing LUTS development were originating from the work environment or the characteristics of the work itself. Therefore, we included nurses who were involved in heavy workplace activities and female civil servants doing desk job working in the same environment. We believe determining the differences between female nurses and civil servants as regards LUTS development together with the problems they experience will be helpful when planning strategic interventions for this problem.

## Methods

### Sampling

This study was designed as a descriptive and cross-sectional survey. It was conducted with nurses and civil servants working at a training and research hospital of a university. The hospital is located at the city center of Ankara province and presents healthcare services to patients with 12 surgical and 15 internal medicine clinics. It also has 13 administrative units in total.

The nurses were selected from the outpatient departments and the surgery, internal medicine, gynecology, emergency and intensive care clinics of the hospital. The civil servants were selected from the administrative (training and courses, organ transplantation, informatics, patient rights, pharmacy, purchasing…..etc.) units in addition to the clinics mentioned above. A total of 406 female staff members are present in the above clinics and consist of 262 female nurses and 144 female civil servants. Male nurses, nurses with a neurological disorder, those with a mental or physical disability or history of a urinary tract infection in the last month; nurses on prenatal, postnatal, breastfeeding, annual or unpaid leave; and those who did not volunteer to participate in the study were excluded. The study was completed with 158 nurses and 105 civil servants who accepted to participate voluntarily between 03 March 2014 and 26 December 2014.

### Data Collection and Instruments

A “Data Collection Form” developed by the investigators after reviewing the relevant literature2,10,15 and the “Bristol Female Lower Urinary Tract Symptoms-Short Form (BFLUTS-SF)” were used to collect the study data. The data collection form included a total of 47 questions on the participants' socio-demographic characteristics (8 questions), history of pregnancy and birth (14 questions), personal hygiene habits (15 questions) and urogynecology history (10 questions). BFLUTS-SF was developed by Jackson et al.^16^ to evaluate urinary incontinence symptoms, other lower urinary tract symptoms, together with their effect on sexual function and quality of life. The questionnaire includes 19 questions and is reported to be a valid and reliable tool with a Cronbach alpha value of 0.78 (16,17). Symptoms related to five areas consisting of storage (question 1–4), urination (question 5–7), urinary incontinence (question 8–12), sexual function (question 13–14) and quality of life (question 15–19) are evaluated with the form. The answers in the questionnaire vary from “never” to “always” and from “no problem” to “serious problem”. No cut off value has been specified in predicting whether LUTS is clinically present in the women completing the BFLUTS-SF. High scores from the questionnaire indicate severe symptoms and increased perceived significance. The Turkish adaptation of the questionnaire was conducted by Güngör & Yalçın18 and cronbach alpha value was found 0.70. Cronbach alpha value of our study was also found to be 0.85.

After obtaining the ethical and institutional permissions for the study, the preliminary administration was performed on 10 subjects who were not included in the study in order to evaluate how clear and practical the data collection tools were. The data collection tools were not changed after the preliminary administration to the nurses and civil servants in their own environment. Completing the data collection tools took 15–20 minutes.

### Data analysis

The analysis of the data was performed using the SPSS 16.0 (SPSS Inc, Chicago, IL, USA) software program. Numbers, percentages, arithmetic means and standard deviation distribution were used in the evaluation of the descriptive data. The presence of a normal data distribution was evaluated with the Kolmogorov-Smirnov and Shapiro-Wilk tests. The comparison of socio-demographic, obstetric, urogynecologic and work-related factors affecting urination between the nurses and the civil servants was conducted with the t test in independent groups and chi-square test. The comparison of the scores obtained by the nurses and civil servants from the BFLUTS-SF was conducted with the Kruskal-Wallis and Mann-Whitney U tests. The statistical significance level was p < 0.05 and the confidence interval was 95%.

### Ethical considerations

Ethical approval (decision no:1491-243-12/1539-541) was received from the Non-Interventional Research Ethics Committee of the university where the study was conducted and written permission was received from the affiliated hospital before starting this study. After the nurses and civil servants received explanation on the aim of the study, their voluntary participation was requested and written informed consent was obtained before the data collection tools were used.

## Results

### Characteristics of nurses and civil servants

The socio-demographic, obstetric and urogyneocologic characteristics of the nurses and civil servants are presented in Table 1. The mean age (33.6±7.1) and body mass index (BMI) (22.5±3.9) of the nurses were found to be significantly lower than the values of the civil servants (38.7±5.4 and 25.4±6.5 respectively) (p<0.05). In the nurse group, 89.2% were university graduates, 59.5% were married and 80.3% stated a BMI of ≤24.9 kg/m^2^. In the civil servant group, 64.8% were university graduates, 68.6% were married and 53.3% stated a BMI of ≤24.9 kg/m^2^. While 57.6% of the nurses included in the study were working in surgical and 24.7% in internal medicine clinics, the majority of the civil servants (59.1%) were working in administrative units. Among the nurses, 64.6% of the nurses expressed that they had cesarean birth. The civil servants' parity was found higher than the nurses; 39.0% of them had two or more parities. A statistically significant difference was found between the nurses and the civil servants in terms of BMI group, educational level, parity and type of delivery (p<0.05). The civil servants (40.3%) were found to have experienced postpartum UI symptoms significantly more commonly compared to the nurses (14.6%) (p< 0.05).

**Table 1:** Socio-demographic, Obstetric and Urogynecologic Characteristics of Nurses and Civil Servants

	Nurses (*n*=158)		Civil Servants (*n*=105)		Statistics	
	
Characteristics	Mean	SD	Mean	SD	t	*p*
**Age (years)**	33.6	7.1	38.7	5.4	-6.21	0.006
**BMI**	22.5	3.9	25.4	6.5	-4.66	0.001

	***n***	**%**	***n***	**%**	***X*^2^**	***p***

**Educational level**						
High school	-	-	28	26.7		
University	141	89.2	68	64.8	47.19	<0.001
Graduate	17	10.8	9	8.6		
**Marital status**						
Married	94	59.5	72	68.6	0.17	0.086
Single	64	40.5	33	31.4		
**BMI groups**						
≤24.9 kg/m^2^	126	80.3	56	53.3		
25.0–29.9 kg/m^2^	22	14.0	31	29.5	21.01	<0.001
30.0–34.9 kg/m^2^	10	5.7	17	16.2		
**Working unit**						
Medical units	39	24.7	12	11.4	82.66	<0.001
Surgical units	91	57.6	27	25.7		
Polyclinics	16	10.1	4	3.8		
Administrative unit	12	7.6	62	59.1		
**Parity**						
None	76	48.1	28	26.7		
One	43	27.2	36	34.3	13.45	0.001
Two and above	39	24.7	41	39.0		
**Delivery method** *						
Vaginal	23	28.1	40	51.9	24.63	<0.001
Cesarean	53	64.6	20	26.0		
Vaginal+ Cesarean	6	7.3	17	22.1		
**UI during pregnancy** *						
Yes	16	19.5	21	27.3	0.33	0.16
No	66	80.5	56	72.7		
**Postpartum UI** *						
Yes	12	14.6	31	40.3	11.95	0.001
No	70	85.4	46	59.7		

Abbreviation: SD, standard deviation; BMI, body mass index; UI, urinary incontinence; t, independent sample- T test; *X*^2^, Pearson chi-square test.

* n_nurses_=82, n_civil ssrvants_=77

The individual habits regarding voiding and work-related factors of the nurses and civil servants are presented in Table 2. No statistically significant difference was found between the nurses and civil servants in terms of delaying voiding, decreasing fluid consumption, not consuming fluid until being thirsty at the workplace (p>0.05). The nurses (60.8%) expressed significantly higher rates of having inadequate time going to the toilet due to their work conditions compared to the civil servants (41.9%) (p<0.05). Adequacy of toilet facility was evaluated as poor and very inadequate by 23.2% of the nurses and 25.7% of the civil servants and a statistically significant difference was present (p < 0.05) (Table 2).

**Table 2:** Voiding habits and work-related factors of nurses and civil servants

Delaying voiding at work	Nurses		Civil Servants		*X* ^2^	*p*

	*n*	%	*n*	%		
Never	18	11.4	10	9.5	2.91	0.40
Sometimes (1 day a week or less)	72	45.6	44	41.9		
Usually (2 or 3 days a week)	37	23.4	21	20.0		
Always (every day or nearly every day)	31	19.6	30	28.6		
**Reduced fluid consumption at** **work**						
Never	51	32.3	35	33.3	0.22	0.97
Sometimes (1 day a week or less)	78	49.4	49	46.7		
Usually (2 or 3 days a week)	23	14.5	17	16.2		
Always (every day or nearly every day)	6	3.8	4	3.8		
**Drinking no fluid until thirsty at work**						
Never	36	22.8	27	25.7	2.78	0.42
Sometimes (1 day a week or less)	72	45.5	47	44.8		
Usually (2 or 3 days a week)	36	22.8	17	16.2		
Always (every day or nearly every day)	14	8.9	14	13.3		
**Adequacy of time for voiding**						
Very adequate	6	3.7	8	7.6	19.44	0.001
Adequate	1	0.6	13	12.4		
Moderate	65	41.2	40	38.1		
Inadequate	32	20.3	34	32.4		
Very inadequate	54	34.2	10	9.5		
**Adequacy of toilet facility**						
Very good	12	7.6	4	3.8	8.53	0.07
Good	61	38.6	52	49.5		
Moderate	50	31.6	22	21.0		
Poor	21	13.3	11	10.5		
Very poor	14	8.9	16	15.2		

Abbreviation: *X*^2^, Pearson chi-square test.

### BFLUTS Scores and Prevalence of LUTs

A statistically significant difference was found between the median scores of the nurses and civil servants from all subscale of the BFLUTS-SF except voiding (p<0.05) (Table 3). The civil servants had higher rates of LUTS related to storage, voiding, incontinence, sexual function and quality of life than the nurses, as seen in Table 4. At least one LUTS was present in 94.2% of the nurses and 97.1% of the civil servants. A statistically significant difference was found between the nurses and civil servants in terms of the LUTS rates, except voiding symptoms and having at least one LUTS (p<0.05).

**Table 3:** BFLUTS Scores in Nurses and Civil Servants

	Nurses		Civil Servants			
	Median	Min-Max	Median	Min- Max	Z*	*p*
BFLUTS-FS	3.00	0.00–9.00	5.00	1.00–10.00	-5.30	<0.001
BFLUTS-VS	1.00	0.00–9.00	1.00	0.00–8.00	-1.51	0.132
BFLUTS-IS	0.00	0.00–13.00	2.00	0.00–14.00	-5.91	<0.001
BFLUTS-Sex	0.00	0.00–4.00	0.00	0.00–5.00	-3.06	0.002
BFLUTS - QoL	0.00	0.00–14.00	2.00	0.00–18.00	-5.56	<0.001
Total BFLUTS	6.00	1.00–40.00	12.50	1.00–46.00	-6.114	<0.001

Abbreviation: BFLUTS, Bristol Female Lower Urinary Tract Symptoms; FS, Filling Symptoms; VS, Voiding Symptoms; IS, Incontinence Symptoms; Sex, Sexual Function Symptoms; QoL, Quality of life symptoms.

* Mann-Whitney U test

**Table 4:** The distribution of LUTS as nurses and civil servants

Symptoms	Nurses		Civil Servants			
			
	*n*	%	*n*	%	*X* ^2^	*p*
**Having at least one LUTS**	149	94.2	102	97.1	-*	0.173
**Filling Symptoms**						
Nocturia (≥2)	39	25.9	58	57.1	25.94	<0.001
Rush to toilet (urgency)	89	60.1	84	81.9	13.94	<0.001
Bladder pain	76	51.0	63	61.0	2.54	0.017
Frequency (≤3 hour between voiding)	73	48.7	65	63.8	5.54	0.023
**Voiding Symptoms**						
Hesitancy	58	39.2	44	42.9	0.29	0.33
Straining	81	54.4	60	59.1	0.74	0.44
Intermittent stream	71	47.5	54	53.3	0.77	0.44
**Incontinence Symptoms**						
Urge incontinence	89	59.5	84	81.9	14.20	<0.001
Frequency of incontinence	36	24.1	36	35.2	3.75	0.037
Stress incontinence	38	25.3	56	55.2	23.80	<0.001
Unpredictable incontinence (no reason and feeling)	19	12.7	27	26.7	8.15	0.004
Nocturnal incontinence	9	5.7	17	16.2	7.69	0.01
**Sexual Function Symptoms**						
Sex life spoiled by urinary symptoms	37	24.7	37	36.3	3.40	0.04
Leak during sexual activity	9	5.7	19	18.1	9.31	0.004
**Quality of Life Symptoms**						
Change outer clothing	18	12.0	28	27.6	10.28	0.002
Cut down fluid	15	10.1	33	32.4	20.29	<0.001
Affect daily tasks	24	15.8	43	41.9	22.17	<0.001
Avoid situations where no toilet	85	57.0	72	70.5	4.90	0.02
Overall interfere with life	27	17.7	43	41.9	18.55	<0.001

Abbreviation: BFLUTS, Bristol Female Lower Urinary Tract Symptoms; LUTS, Lower Urinary Tract Symptoms; *X*^2^,Pearson chi-square test.

* Fisher's Exact Test.

### Risk Factors of LUTs

The intergroup and intragroup comparison of the socio-demographic, obstetric and urogynecologic characteristics of the nurses and civil servants according to the total BFLUTS-SF scores is presented in Table 5. When the intergroup differences median scores from the BFLUTS-SF scores in terms of age, BMI, educational status, medical and surgical working unit, parity, having cesarean and vaginal delivery and urinary incontinence in their previous pregnancies were compared between two groups, statistically significant differences were found (p<0.05).

**Table 5:** Comparison of in-groups and intergroup of Socio-demographic, Obstetric and Urogynecologic Characteristics of Nurses and Civil Servants as Total BFLUTS-SF Scores

Characteristics	Total BFLUTS-SF Scores		Z**	*p*

	Nurses	Civil Servants		

	Median (min–max)	Median (min–max)		
**Age**				
21–34	5.00 (1.00–32.00)	10.00 (1.00–24.00)	-2.795	0.005
35–52	7.00 (1.00–40.00)	14.00 (1.00–46.00)	-4.329	<0.001
	*Z*=-2.70, *p*=0.007	*Z*=-554.0, *p*=0.007		
**BMI**				
≤24.9 kg/m^2^	6.00 (1.00–32.00)	9.00 (1.00–46.00)	-3.357	0.001
25.0–29.9 kg/m^2^	9.00 (1.00–26.00)	14.00 (2.00–28.00)	-2.603	0.009
30.0–34.9 kg/m^2^	7.00 (1.00–40.00)	18.00 (4.00–40.00)	-2.092	0.030
	X^2^*=3.67, *p*=0.160	*X^2^*=22.25, *p*<0.001		
**Educational level**				
High school	-	15.00 (1.00–29.00)	-	-
University/Faculty	6.00 (1.00–40.00)	13.00 (2.00–46.00)	-4.921	<0.001
Graduate	5.00 (1.00–17.00)	11.00 (3.00–13.00)	-1.113	0.280
	*X^2^*=-0.62, *p*=0.532	*X^2^*=3.37, *p*=0.185		
**Working unit**				
Medical units	7.00 (1.00–21.00)	15.00 (3.00–28.00)	-3.070	0.020
Surgical units	6.00 (1.00–40.00)	18.00 (1.00–28.00)	-5.062	<0.001
Policlinics	7.00 (1.00–26.00)	9.00 (5.00–29.00)	-0.853	0.446
Administrative unit	6.00 (2.00–14.00)	10.00 (1.00–28.00)	-2.219	0.026
	*X^2^*=-3.10, *p*=0.377	*X^2^*=-8.48, *p*=0.037		
**Number of childbirths**				
No child bearing	7.00 (1.00–32.00)	11.00 (1.00–24.00)	-2.731	0.006
One birth	6.00 (1.00–18.00)	10.00 (2.00–28.00)	-2.828	0.005
Two or more births	5.00 (1.00–40.00)	20.00 (1.00–46.00)	-4.543	<0.001
	*X^2^*=1.43, *p*=0.489	*X^2^*=10.65, *p*=0.005		
**Delivery method**				
Vaginal	6.00 (1.00–40.00)	10.00 (1.00–40.00)	-2.099	0.036
Cesarean section	5.00 (1.00–23.00)	7.00 (2.00–18.00)	-1.63	0.102
Vaginal and cesarean	5.00 (1.00–9.00)	23.00 (5.00–46.00)	-3.412	0.001
	*X^2^*=2.70, *p*=0.259	*X^2^*=17.26, *p*<0.001		
**UI during pregnancy**				
Yes	13.50 (4.00–40.00)	18.00 (4.00–46.00)	-1.530	0.126
No	5.00 (1.00–16.00)	10.00 (1.00–29.00)	-5.100	<0.001
	Z=-4.39, *p*<0.001	Z=-2.70, *p*=0.007		
**Postpartum UI**				
Yes	17.00 (4.00–40.00)	23.00 (4.00–46.00)	-5.100	<0.001
No	5.00 (1.00–16.00)	9.00 (1.00–28.00)	-4.127	<0.001
	Z=-4.58, *p*<0.001	Z=-2.63, *p*<0.001		

Abbreviations: BFLUTS-SF, Bristol Female Lower Urinary Tract Symptoms Short Form; UI, Urinary Incontinence; min; Minimum; Max, Maximum,

* Kruskal-Wallis test

** Mann-Whitney U test

## Discussion

LUTS are common in women and can be experienced differently in the daily lives by the women.^1^,^11^,^19^ Nurses and civil servants commonly experienced LUTS especially urgency, urge incontinence, avoiding places with no toilet, straining to urinate, and having to urinate after less than three hours in our study. An another study on nurses and secretaries in Turkey emphasized the most common LUTS to be stress incontinence, urgency and urge incontinence^20^. These different results could be due to the differences in data collection tools used to determine LUTS and the study populations. The prevalence varies according to the place the study is conducted, the study population and the form of data collection.^4^,^5^,^6^,^7^ This study showed that the prevalence of at least one LUTS was very high in nurses and civil servants (94.2% and 97.1% respectively) and there was no difference between the nurses and civil servants in terms of the LUTS prevalence rates. Similarly, Kaya et al.^19^ did not report a difference between nurses and secretaries in terms of the prevalence of LUTS in their study. The LUTS prevalence in nurses and civil servants in our study was higher than the other studies.^2^,^19^,^20^,^21^ The prevalence of LUTS is reported to increase with working conditions in the literature^2^,^13^,^14^. This could explain the higher prevalence of LUTS in our study.

Working conditions have been reported to potentially influence the development of LUTS^2^,^14^,^15^. Environmental factors at the workplace and working status^13^ and the bladder discharge habits of the individuals^22^ have been reported to be effective in the development and worsening of LUTS. The percentage generally or always delaying voiding while at the workplace was 43.0% in nurses and 48.6% in civil servants in our study. The percentages of delaying voiding at the workplace in our study is similar to those reported by two studies.^13^,^19^,^23^,^24^ The other study emphasized that the nurses have difficult working conditions and an irregular lifestyle, necessitating often delaying voiding.^20^ Our study found that the nurses evaluated the duration of toilet breaks as very inadequate and adequate at significantly higher rates than the civil servants. Similar result was also revealed by other study.^2^

Nurses are reported to suffer higher work-related and physical stress than the normal population.^20^ However, the LUTS items except voiding symptoms were found to be significantly more common in civil servants who are thought to have a sedentary lifestyle and not perform challenging activities compared to the nurses. Sedentary lifestyle can increase risk of urinary tract symptoms. Sedentary lifestyle (prolonged sitting and physical inactivity) can produce changes in the musculoskeletal and vascular milieu in the pelvis and lower extremity vasculature and these changes may cause LUTS.^25^

In the current study, we found that development of LUTS were affected by age, BMI, number of childbirths, delivery methods, and having UI during pregnancy and postpartum. It is known that the prevalence of developing LUTS increases with advancing age.^2^,^8^ The median total scores from the BFLUTS-SF increased in both the civil servants and nurses with increasing age in this study. This result was similar to those of Liao et al.^2^ and Zhang et al.^3^ Increased BMI, a well-known risk factor for LUTS in both females and males, was reported to have a significant relationship with LUTS.^26^ In the current study, median BFLUTS-SF scores of civil servants increased significantly with BMI. A significant difference between BMI and LUTS was not reported in a study on nurses in China.^7^ Having given birth and especially vaginal birth is reported to be a risk factor for LUTS development.^4^ The median BFLUTS-SF score was lower in nurses and civil servants who gave birth with cesarean. Similarly, cesarean birth was found to be protective regarding LUTS development in the study conducted by Wan et al.^21^ Increasing parity meant a significant increase in the median BFLUTS-SF score in only civil servants in our study. A significant relationship was found between having given birth and the incidence of at least one LUTS in nurses in the study of Zhang et al.^20^ Literature reported that 4 or more pregnancies and births in incontinent women markedly raised the median BFLUTS-SF score but no statistically significant difference was found between parity and BFLUTS scores in continent women.^18^ The risk of developing UI has been reported tincrease with a history of UI symptoms in previous pregnancies and the postpartum period^27^,^28^. A statistically significant relationship was found between the presence of UI symptoms during pregnancy and the postpartum period and the median total scores from the BFLUTS-SF score when the nurses and civil servants were compared among themselves and with each other. Our study results are consistent with literature. 27,28

This study had certain limitations. One of the limitations is that the study sample was selected from a single hospital in Turkey. Other limitations are the sectional characteristic of the study and the data on LUTS being based on the subjective information provided by the individuals. And the last one is that being a health worker is an occupation subject to a high degree of stress, and stress levels of nurses and civil servants and effects of stress on LUTS of them weren't measured in this study.

## Conclusion

The prevalence of at least one LUTS in nurses and civil servants was found to be high in this study. The nurses decreased their numbers of voiding significantly compared to the civil servants due to their intensive workload. We also found that advanced age and having UI during pregnancy and postpartum period played a role in the LUTS prevalence in both professional groups. The working conditions of nurses and civil servants at their workplace affected their voiding behavior. We believe reorganizing the work environment and conditions for the employees to have healthy bladder habits at the workplace is important for preventing the development of LUTS and decreasing the recurrence and severity of existing symptoms. It would therefore be useful to investigate what the urinary habits of nurses and other staff at home, at work, or in the society mean for them in order to help them develop healthy behaviors.
